# Understanding the Complexities of Eliminating Trans Fatty Acids: The Case of the Trans Fatty Acid Ban in Thailand

**DOI:** 10.3390/nu14132748

**Published:** 2022-07-01

**Authors:** Weerapak Samsiripong, Sirinya Phulkerd, Umaporn Pattaravanich, Manasigan Kanchanachitra

**Affiliations:** Institute for Population and Social Research, Mahidol University, Phutthamonthon, Nakhon Pathom 73170, Thailand; s.weerapak@gmail.com (W.S.); umaporn.pat@mahidol.ac.th (U.P.); manasigan.kan@mahidol.edu (M.K.)

**Keywords:** food policy, trans fatty acids, obesity, noncommunicable diseases, Thailand

## Abstract

Trans fatty acids (TFAs) have no known health benefits and are linked to an increased risk of noncommunicable diseases (NCDs). To eliminate TFAs from the food supply, the government of Thailand imposed a ban on partially hydrogenated oils (PHOs) in 2018. This study was aimed at analyzing the government policies and actions to eliminate TFAs in Thailand, focusing on policy content, context, process, and actors. This single-case qualitative study used a documentary review and interviews with 20 key policy actors. The data analysis was guided by thematic analysis based on the policy triangle framework. The results reveal that policy actors—government organizations, academics, civil society organizations, and the food industry—have different roles, interests, and influences with regard to eliminating TFAs in Thailand. Both formal and informal communication among policy actors aided in the policymaking process and the actions that followed. Changing perceptions of TFAs, the low intake of PHOs in Thailand, hype around trans fats, and trade dynamics shaped the government’s decision to impose the ban. As a result, the ban was selected to avoid the technical components of TFA elimination. This study suggests that eliminating TFAs in Thailand could be further enhanced by strengthening government actions in terms of enforcement and creating consumer awareness.

## 1. Introduction

Trans fatty acids (TFAs) are unhealthy nutrients consumed worldwide. TFAs have no known health benefits [1] and are linked to an increase in low-density lipoprotein (LDL) and a higher risk of cardiovascular disease and other noncommunicable diseases (NCDs) [2,3,4,5]. Consequently, approximately 540,000 global deaths from coronary heart disease (CHD) are attributed to TFAs every year [6]. Regardless of their adverse health impacts, TFAs are still present in the food supply. The intake of TFAs ranges from 0.2 to 6.5% of total energy intake for adults globally [7]. The adverse health impacts of TFAs and the uneven distribution of TFA consumption across countries have triggered global efforts to virtually eliminate TFAs from the food supply.

Eliminating TFAs has been a policy priority of the World Health Organization (WHO) [8]. The WHO’s 13th General Programme of Work (2019–2023) recommended the elimination of industrially-produced trans fatty acids (IP-TFAs) to reduce the burden of NCDs [9]. In May 2018, the REPLACE action package, which constitutes six strategic action areas—review, promote, legislate, assess, create, and enforce—was introduced to provide a roadmap for national actions to eliminate TFAs from the global food supply by 2023 [10,11].

The best-practice policies of REPLACE include a legislative limit of 2 g of TFAs/100 g of total fats and oils in all foods and a ban on partially hydrogenated oils (PHOs). Examples of nations taking action that provide best-practice cases using these two approaches include Denmark and the United States. Denmark was the first country to impose a limit on TFAs per 100 g of fat or oil in 2003, which led to a reduction in the mortality rate from CHD by 4.3% [12,13]. The United States, on the other hand, imposed a PHO ban by announcing its removal from Generally Recognized as Safe (GRAS) food products in 2015, which came into effect in 2020 [14]. It was expected that the REPLACE action package would provide momentum to eliminate TFAs in 40 countries by 2022. However, despite numerous research studies supporting the elimination of TFAs, the adoption of relevant policies is concentrated in higher- or upper-middle-income countries [15].

Thailand provides an important case study for TFA elimination, as it was listed as 1 of the 15 countries where REPLACE best-practice policies were adopted in 2020 [10]. The intake of TFAs in Thailand had been in the range of 0.75 to 0.99% of total energy intake for adults. Although TFA intake in Thailand was already below the FAO/WHO’s recommendation (<1% of total energy intake), TFAs were still present in some food products. TFAs constituted 15–43% of the total weight of margarine and shortening with PHOs. The use of PHOs in food preparation and processing can lead to higher TFA content in food items, such as baked goods (2.2% of the total weight) [7].

In eliminating TFAs, the Food and Drug Administration (FDA) of Thailand co-operated with the Institute of Nutrition (INMU) to launch the Thailand: Trans Fat-Free Country project as a situational analysis and a platform for a stakeholder meeting with the food industry in 2016. In 2018, the FDA announced Ministry of Public Health (MOPH) Notification No. 388 Re: Prescribed Prohibited Food to be Produced, Imported, or Sold, which came into effect on 9 January 2019. Despite the government’s progress in TFA elimination, analyses of TFA elimination policies in Thailand are limited [16,17,18]. Previous research was based primarily on the scientific analysis of TFAs in the food supply, while the public policy process that shed light on their elimination was underexplored.

As a public policy, the policy to eliminate TFAs in Thailand has underlying driving forces of content, context, process, and actors. This paper aims to analyze government policies and actions to eliminate TFAs in Thailand, focusing on policy content, context, process, and actors, to unravel the challenging complexities and the opportunities for improvement. The findings of this study represent a lesson learned for implementing TFA elimination policies in countries with similar contexts, especially in southeast Asia.

## 2. Materials and Methods

This research is a single-case qualitative study. It employed a case study approach to study TFA elimination policies in Thailand. This is a research approach that provides insight into complex issues in a real-life context [19]. The single-case design can help explain how critical, unusual, revelatory, or longitudinal a case can be [20]. To unravel the complexities of TFA elimination in Thailand as a case study, qualitative research was used in data collection and analysis.

The analysis of government policies and actions was underpinned by the policy triangle framework [21]. The policy triangle framework posits that the field of public policy is contested by various actors within the complexities of content, context, and process. Within this framework, conventional policy analysis that puts an overwhelming emphasis on policy content is criticized for undermining the process, context, and actors that might have an impact on success or failure [21]. The concepts explored in the interviews are given in Table 1.

### 2.1. Data Sources and Collection

Data were collected using a documentary review and in-depth interviews. The Institutional Review Board, Institute for Population and Social Research, Mahidol University (IPSR-IRB), granted approval to conduct the study (COA no. 2021/07-147).

A documentary review was conducted using a systematic search in academic databases (SCOPUS and Thai Journals Online). To complement the analysis, a database search of institutional websites and news archives, as well as a Google search, were conducted to ensure the comprehensiveness of the documentary sources. The references in the documents underwent snowball sampling. Documents were included if they were (1) relevant to public policy and (2) relevant to eliminating TFAs in Thailand. The data were stored as a package of case records concerning existing government policies and actions to eliminate TFAs in Thailand based on content, context, process, and actors. The description of documents in an analysis of the PHO ban in Thailand is indicated in Table 2.

In-depth interviews with stakeholders were conducted from September 2021 to January 2022. Forty-one stakeholders from government organizations, academic institutions, civil society organizations, and the food industry were invited, and 20 of them participated in the study (Table 3). The selection of stakeholders was primarily based on named-entity recognition in the news archives. The identification of stakeholders was augmented by a purposive snowball sampling strategy [4]. The duration of the interviews was 60–120 min. Informed consent was obtained from all subjects involved in the study. The participants’ identities were anonymized with the labels GO1-9 for the representatives of government organizations from various offices, departments, and ministries from the central administration in Thailand, as well as an international governmental organization; AC1-5 for academics from various academic institutions specializing in nutrition, food science, and health policy; CS1-4 for the representatives from non-state, not-for-profit civil society organizations in the areas of public health nutrition and consumers’ protection; and FI1-2 for the representatives from the food industry with the stakes on with TFA elimination.

### 2.2. Data Analysis

The data analysis was guided by thematic analysis. Thematic analysis is a method that helps to understand themes (patterns of meaning) that emerge across datasets based on shared meanings or experiences [5]. The interviews were recorded and transcribed verbatim. The data were coded in line with the policy triangle framework using NVivo software by 2 main researchers (WS, SP). Additional codes were added from open coding to ensure the comprehensiveness of the assigned codes. To ensure the credibility of the codes, all authors were assigned a sample of transcripts to code, and intercoder differences were considered. The interpretation of themes identified in the coded data was clarified through discussion among all authors.

## 3. Results

This section is divided into four main sections: policy content, policy context, policy process, and policy actors.

### 3.1. Content

The policy content was analyzed based on how the policy was problematized and how controversies arose from selecting a specific policy problem. In Thailand, the PHOban was selected as a solution to avoid the technical components of problematizing TFA elimination, though it was criticized for limited traceability and a lack of consumer engagement.

Many actors believed that eliminating TFAs was technical. In explaining what TFA elimination was, many participants discussed the complexity in the definition of TFAs; as one academic explained, “trans fats may naturally occur or be derived from partially hydrogenated oils” (AC3). Many participants agreed that eliminating TFAs as a nutrient was difficult because “there is no way to remove naturally occurring trans fats” (FI1), and “if we ban trans fats directly, it means that we might not be able to drink milk because trans fats naturally occur in these products” (GO1), as reported by participants from the food industry and a government organization, respectively.

To avoid the technical component of TFA elimination, the PHO ban was selected as a problem-solving mechanism to minimize potential public controversies under budget constraints; as one participant noted, “We approached it this way because we did not want to bother the citizens. The public sector could monitor this based on the available budget” (AC1).

Although the PHO ban was selected to avoid the technical component of TFA elimination, many participants reported that TFA elimination in Thailand was not free of controversy. Many participants expressed doubt about the effectiveness of the PHO ban because of limited consumer engagement and traceability. In terms of consumer engagement, a participant from the food industry stressed the importance of disseminating knowledge to consumers: “When the law is legislated, the knowledge should be disseminated along with it” (FI2).

Furthermore, some participants questioned the traceability of the policy and called for cyclic monitoring, a mechanism to “assess the content of trans fats in Thai diets with an indicated time” (GO2). Limited traceability and consumer engagement pushed government authorities to bear the responsibility for the policy’s enforcement mechanism, which was highly technical; one government representative commented:

“The citizens have no way to know. There is no way the citizens can collect samples and submit them for analysis to identify whether or not the food is safe. It is all the responsibility of the public sector, but the public sector did not perform this role” (GO2).

### 3.2. Context

Policy context was analyzed in four dimensions (historical, political, economic, and sociocultural). This section explains how the government’s decision to impose the PHO ban was shaped by changing perceptions of TFAs, the low intake of PHOs in Thailand, trans fats hype, and trade dynamics.

A review of documents and interviews with policy actors indicated changes in perceptions of TFAs throughout history. One document noted that “PHOs became common in bakery products in Thailand beginning in 1977” (RES3). PHOs were used because of their desirable characteristics; one civil society participant commented that “they created a crispy texture, delicious taste, and flavor in food, and they could be kept at room temperature without going rancid” (CS2). However, later, TFAs were considered unhealthy because they “increase LDL, the bad fat” (FI1). Because they are unhealthy, many participants explained that TFAs were replaced with other alternatives, including full hydrogenation.

However, a participant from the food industry commented that full hydrogenation created “undesirable characteristics” because “the fats would be solid and not spreadable” (FI1). Therefore, another alternative, oil blending, was used in the food industry; an academic participant explained that “now, we have blended oils. We blend soybean oil with palm oil in proportion. It is a multipurpose oil accessible by all and locally available” (AC1).

In terms of the sociocultural context, PHOs are not commonly used at the household level, based on the documents and interviews with policy actors. One document noted that “Thailand is blessed with naturally saturated tropical oils, some of which have been used for generations” (RES2). Therefore, given the availability of other oils, PHOs have been “rarely used in local Thai cuisines” (RES2). Most participants supported this argument; one academic discussed how consumption in Thailand shaped the government’s decision to impose the PHO ban: “Our country had saturated fats from coconut oils and palm oils by nature, and they were cheap” (AC1).

Regarding the political context, policy actors discussed the waves of hype around trans fats that influenced the government’s decision to impose the PHO ban. Some recalled the earlier wave of hype in Thailand that was triggered by the enactment of TFA labeling regulations in the US in the early 2000s; an academic explained that “trans fat labeling regulation in the US initiated the Thai government’s response to banning trans fats” (AC1). However, government actions on TFA elimination were opposed because “it would not yield any results” (AC1).

Policy actors recalled another wave of trans fat hype in Thailand that reappeared on the policymaking agenda in 2015 because “bans were imposed in the US and Europe” (AC1). This situation triggered the government’s decision to impose the PHO ban because of the potential TFA influx in Thailand; as one academic explained, “Trans fats produced in large quantities would be sent from these countries [the US and Europe] to replace our available oils with a competitive price advantage because they could not be sold in their countries” (AC1).

In terms of economics, some participants connected the role of Thailand as a producer of palm oil with the aspiration of product reformulation. A document reported that “Thailand is a palm oil producer” (RES2). An academic further explained that “our country is a food export country. Therefore, the presence of trans fats in our food exported is already avoided” (AC1). Some participants believed that market competition created an environment in which food safety is of utmost concern, which is a self-regulating principle in the food trade. As a result, imposing the PHO ban in Thailand would generate economic benefits; one participant from the food industry reported that “when the law is enforced, it creates 100% confidence that there must not be anything related to the occurrence of trans fats in the production process” (FI2).

### 3.3. Process

The policy process was analyzed based on how a policy was made and how it fared in the implementation. It was found that formal communication through research collaboration and informal communication among policy actors facilitated the policymaking process, and the policies and actions to eliminate TFAs were prevalent shortly after Notification No. 388 was announced.

Policy actors explained how research collaboration created a platform for formal communication. The FDA collaborated with academics to launch the Thailand: Trans Fat-Free Project. Throughout the project, several meetings were held in 2017 that were attended by many participants in order to “devise strategies for TFA elimination” as indicated in a document (RES4). Policymakers were invited so that “they would see the significance as to why it had to be notified and what details of the notification were” (AC3), especially throughout the prevailing consumer hype around trans fats at the time. As a result, the political negotiation was successful because “he [the policymaker] weighted the impacts on the small group of producers against the votes gained” (CS2).

Many participants from the policy sector mentioned the informal communication among policy actors that facilitated the legislation. One government representative noted that “the academic sector produces personnel for the private sector” (GO2). This relationship created an informal relationship with the food industry, where “calls were frequently made to receive feedback on the policy” (GO2). Despite the relationships among actors, there were suspicions among actors in the formal platform; one academic reported that “they did not trust us because we were going to impose rules on them” (AC3). However, the frequent meetings mitigated suspicions among actors, and ties with the food industry were strengthened; one academic reported that “the food industry made frequent calls to inquire whether they can submit products for analysis” (AC2).

Many participants discussed the government imposing control over the food industry during the grace period. The grace period was considered a “preparation period” (GO4), which allowed the food industry to “make changes without being immediately punished by law” (GO4), as indicated in Notification No. 388: “this notification shall come into force after 180 days from the date of its publication” (DOC1). This period allowed the government actors to “notify the manufacturers” (GO4) and the food industry to clear production lines that were “planned a year in advance” (FI2). Though the proposed grace period was one year, it was shortened to 6 months to keep up with the ongoing global situations “as demanded by the oil producers because they were afraid of an influx of oils with trans fats from other countries” (AC1).

To ensure compliance with the law, random inspections and document reviews were used as primary methods of policy implementation, as indicated in the implementation manual of Notification No. 388: “Inspection and monitoring activities occurred at the producing, importing, and distribution facilities based on the consideration of documents […] and random inspection” (DOC3).

Based on the aforementioned mechanism, many participants felt that TFA elimination in Thailand was already finished or less prioritized. One government representative reported that further actions on TFA elimination might not be necessary because “many issues can be proposed, but it might not be feasible or cost-effective because it cannot do anything much” (GO1). However, many felt that there should be further post-monitoring activities. The limited actions following the enactment of Notification No. 388 cast doubt among those with public health interests; one academic explained:

“I feel that we have a good ascending phase, meaning the policy was issued, but for the implementation. […] I do not see how the industry reformulated or how the enforcement was carried out” (AC4).

### 3.4. Actors

The analysis of policy actors was based on their different roles, interests, and influences with regard to TFA elimination. The results revealed that the FDA performed its role as both a regulator and a buffer organization in co-operating with the academics, civil society organizations, and food industry personnel. Other key actors included the INMU, which provided technical guidance, and the Foundation for Consumers, providing policy communication. The role of the food industry in pushing forward changes in TFA elimination was also highlighted.

Laws provide authority to government organizations in TFA elimination. An analysis of the documents revealed that TFA elimination involved various laws: “Thai laws that can be used to control trans fats in food include the Food Act B.E.2522; the Consumer Protection Act, B.E.2522; Export and Import of Goods Act, B.E.2522; National Food Committee Act, B.E.2551; and Agricultural Standards Act, B.E.2551” (RES1).

These laws allowed the FDA to have power as the regulator of TFA elimination. However, this regulatory role was limited in many regards. A participant from a government organization pointed out that there was an emphasis on “packaged food” (GO1). Furthermore, some participants further explained that the FDA’s role was limited by budget allocation for each nutrient; an academic reported that “they have the budget for the routine activity, but there are so many missions” (AC1). To overcome the limitations, the FDA co-operated with actors with a stake in TFA elimination, particularly the food industry, civil society organizations, and academics; as one government participant explained, “The FDA had an engagement from stakeholders or involved parties—the consumers, specialists, academics, or institutions—to provide information; the food industry was engaged to foresee the feasibility” (GO8).

Although the engagement of policy actors eased the FDA’s limitations, they expressed many different beliefs, creating a cacophony around TFA elimination in Thailand. As a result, the FDA acted not only as a regulating authority but as a buffer organization in TFA elimination; as one academic explained: “The FDA is a buffer organization. They have to put up with resistance from the industries and forces from the civil society organizations and the academics” (AC4).

Most participants believed that the engagement of academics was integral in the policy process. However, some believed that academics should “only produce evidence to the regulator or involved parties” (AC4) and “do not advocate for policies to avoid potential conflict of interests” (AC4). However, many refuted this idealism; one academic explained the importance of academic advocacy:

“We [academics] have to prove that we would be with them until the end, not just pitch the idea and let them [governmental organizations] get attacked. After-sales service was important” (AC1).

Among the academics, some praised the contributions of the INMU to TFA elimination. They explained that the institute “was a keyman […] who worked with us because we had to think about who and how [TFA elimination] should be” (GO1) and “helped in the collection of samples and technical details” (CS1), in the words of participants from a government organization and a civil society organization, respectively.

Some participants believed that the engagement of civil society organizations was significant in TFA elimination. While some from civil society organizations expressed an interest in consumer protection, especially regarding the “possible presence of trans fats in Thai diets” (CS1), others had an interest in “promoting nutrition literacy” (CS2) and emphasized that “people need to be literate about whether trans fats are present in the products” (CS2).

Participants reported that the Foundation for Consumers was the most influential among civil society organizations with regard to TFA elimination, especially in “monitoring the situation” (AC1) by “conducting a random inspection” (GO2). Some explained that the Foundation for Consumers used this information for “public disclosure” (AC1), which could be used as “policy communication to the public” on the one hand and as a mechanism to ensure compliance from the food industry on the other; as an academic explained, “The food industry would beg for the information not to be released, and they would eliminate them [TFAs] in return” (AC1).

Many participants discussed the food industry’s engagement in TFA elimination. Although the food industry’s engagement in the policy process was to secure trade interests by ensuring that “the laws legislated protect both consumers and the industries” and “no industries die out from policy change” (FI2), the food industry could be categorized into two factions, complying and non-complying industries (AC1).

Some participants in the policy sector believed that the food industry has the capacity to make voluntary changes, especially “local oil manufacturers” (AC1), because they can “solve problems by using palm oils or blended oils to reduce trans fats” (AC1). However, there are also “multinational companies” that do not “make changes unless a law is legislated” (AC3). In this sense, many participants claimed that TFA elimination should be pushed forward as a law to ensure the survival of complying industries; as one participant from a civil society organization explained,

“We create fairness for good industries that are concerned about the consumers. If we do not regulate, the bad industries would proliferate, and the food industries will die out. We do not want to see that picture” (CS4).

## 4. Discussion

This research was the first effort to analyze the government’s policies and actions to eliminate TFAs in Thailand. Underpinned by the policy triangle framework, the complexities of these policies and actions were analyzed based on content (problematization and controversy), context (historical, political, economic, and sociocultural), process (policymaking and implementation), and actors (roles, interest, and influence). The analysis of policy content, context, process, and actors revealed two significant findings: perceived policy success (political economy of TFA elimination, policy quiescence in the decision-making process, and multisectoral engagement in TFA elimination) and perceived implementation gaps (limited policy continuity and engagement of consumers).

**Figure 1:**
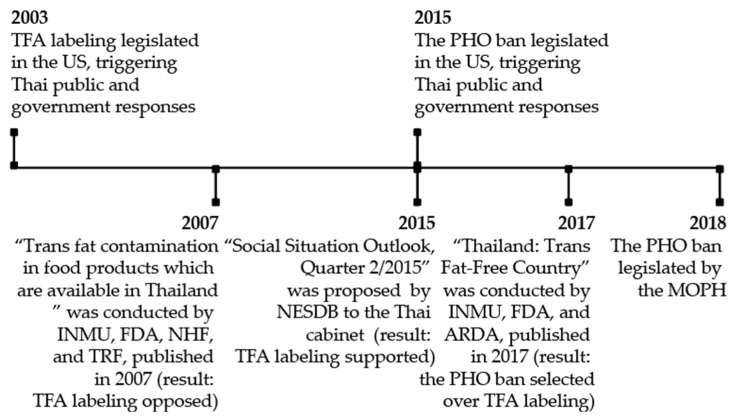
Timeline of TFA elimination policy decisions in Thailand.

The perceived policy success in Thailand underwent a long policy process. Policies legislated in the US (TFA labeling in 2003 and the PHO ban in 2015) triggered Thai public and government responses to TFA elimination. Although TFA labeling was proposed as a mechanism to eliminate TFAs in Thailand, it was opposed by the INMU, the FDA, National Health Foundation (NHF), and Thailand Research Fund (TFA) through a research collaboration on Trans Fat Contamination in Food products Which Are Available in Thailand [26] and resurfaced to the decision-making arena in 2015 by the National Economic and Social Development Board [27]. Both policies were reviewed by policy actors in the Thailand: Trans Fat-Free Country conducted by the INMU, the FDA, and the Agricultural Research Development Agency (ARDA) [28]. As the PHO ban was selected over TFA labeling, the PHO ban was legislated by the MOPH. Figure 1 indicates the timeline of TFA elimination policy decisions in Thailand.

One major driving force attributed to the PHO ban being adopted in Thailand was the political economy of TFA elimination. While the ban fulfilled the government’s political interest in responding to the domestic hype and global efforts to eliminate TFAs, it also fulfilled the trade interests of the food industry in domestic food governance and global trade. The political economy of trade and law was also contributive to nutrition and alcohol policymaking in South Africa and was noted as a potential contributing factor to TFA elimination in Cambodia [14,29].

A state of policy quiescence was attained in the communication process when adopting the PHO ban in Thailand. Compared to other public health and nutrition policies, specifically obesity policies, which generally became an issue with policy actors sifting through a cacophony of competing evidence and claims from stakeholders and public opinions [30], in Thailand, policy quiescence was achieved through formal and informal communication. Informal communication comprised a prolonged and hidden process in which policy actors negotiated their interests, advocated for the policy, and framed specific actions from behind the scenes. Regardless of the pre-existing informal communication, doubts arising from the differences in roles, interests, and influence among policy actors were prevalent. Therefore, the Thailand: Trans Fat-Free project was a research program that provided a platform on which policy actors could reach a certain level of agreement, resulting in the legislation of Notification No. 388.

Engagement by policy actors was one factor contributing to the adoption of a TFA elimination policy in Thailand. As both the regulator and a buffer organization, the FDA overcame its institutional limitations by fostering engagement among academics to provide clarity on the technical components of TFA elimination, the food industry to ensure policy support, and civil society organizations to ensure policy communication. The engagement of policy actors was noted as a key component in accelerating policymaking and implementation, as was the case in India, where the need for multisectoral food chain approaches to reduce TFA consumption was recognized, but none had yet been applied [5,31]. Therefore, the policy content of the PHO ban reflected a public policy in which the technical components of TFA elimination were compromised by the political economy, policy quiescence in the decision-making process, and multisectoral engagement.

The perceived implementation gaps in Thailand could be identified by contrasting policies and actions held in Thailand against policies and actions in other countries and the WHO’s REPLACE action package. Thailand implemented the PHO ban, which corresponded with the recommendations in the Legislate module of the WHO’s REPLACE action package, which indicated two policy options (a mandatory limit on the amount of TFAs in all foods or the PHO ban). The selection of the policy was slightly different from those of the US whereby the PHO ban was implemented by the federal government following TFA labeling policy, supported by the implementation of policies at the state level [32,33]. Thailand’s policies and actions were entirely different from those implemented in Denmark, whereby the legislative limit of TFAs was imposed following TFA labeling policy [12,34]. Although the policies and actions in Thailand corresponded with the WHO’s REPLACE action package, the absence of TFA labeling policy in Thailand became the source of the perceived implementation gaps among policy actors.

The findings reveal that the perceived implementation gaps addressed by the policy actors include policy continuity and consumer engagement. Policy continuity was an area that needed further government policies and actions. Many study participants explained that there were limited actions after the policymaking process. As TFA elimination was legislated by Notification No. 388, policies and actions were based on legal interpretations and relied heavily on government organizations dealing with a myriad of responsibilities under budget constraints. A lack of continuity has been noted as an issue in the implementation of food and nutrition policies in many countries, such as Colombia and Spain [35,36]. To ensure continuity, the Chilean government made an agreement with the academic sector to ensure a broad consensus and continuity over time [37]. In Thailand, policy continuity for TFA elimination was secured since it was legislated into law, though this does not guarantee that there will be further research or budget support for post-marketing activities.

Furthermore, as TFA analysis is highly technical, the role of consumers in eliminating TFAs has been limited. Many countries have engaged consumers by including TFAs on the nutritional facts panel. However, the impact of the halo effect, whereby consumers might misinterpret nutritional information and make misinformed purchasing decisions with regard to products with health or nutrient content claims, such as being TFA-free, has been noted [38,39]. In Thailand, the halo effect was avoided because TFA labeling was not encouraged and TFA-related claims were not permitted. However, the engagement of consumers was still an issue that needed further attention.

The lesson drawn from the analysis of the government’s policies and actions to eliminate TFAs in Thailand based on content, context, process, and actors was that the government and involved parties made swift progress by announcing Notification No. 388 in 2018, which prohibits the production, distribution, and import of PHOs, as a response to changing historical, sociocultural, political, and economic contexts. However, as TFA elimination was a work in progress, Thailand needed to adopt a comprehensive approach by raising consumer awareness of TFAs and strengthening the enforcement mechanisms to ensure policy continuity. In addition, to ensure that Thailand achieves global targets for TFA elimination, further research should be conducted to assess the government’s progress.

This study has limitations. This research used a case study approach to analyze government policies and actions to eliminate TFAs in Thailand. This research might be transferable to other upper-middle-income countries with a similar policy context, where the intake of TFAs is generally low. Further research in the context of lower-middle-income countries might be needed. Furthermore, this research employed a qualitative method to analyze policy actors’ views on government policies and actions to eliminate TFAs. Policy actors from various sectors were recruited in this study in order to gain insight into TFA elimination in Thailand. There were fewer representatives from the food industry. The recruitment of the participants from the food industry sector was limited due to the bureaucratic structure in Thailand. The representatives of the food industries in Thailand in the policy process were generally recruited from organizations with the authority to be a representative of the food industry in forging public-private co-operation indicated by law. Therefore, the individual food and beverage industries were less inclined to respond to the interview invitation. Furthermore, although PHOs were eliminated from the food supply following the legislation of Notification No. 388, some policy actors from the food industry were concerned about the circulation of the misconceptions regarding the presence of TFAs in the Thai food system, which might allow another wave of trans fat hype to resurface. In this respect, the perspectives from this sector might not be comprehensively represented. This research used the perceived roles, interests, and influence of policy actors regarding the food industry to mitigate this limitation. Furthermore, translating the interviews posed a linguistic challenge in data analysis and reporting, especially with regard to tenses and unclear antecedents. The analysis was based on the original Thai transcripts to mitigate the linguistic challenge, and back translation was used to ensure that the participants’ sentiments were accurately represented.

## 5. Conclusions

Underpinned by the policy triangle framework, this qualitative case study analyzed government policies and actions to eliminate TFAs in Thailand based on content, context, process, and actors. Policy actors had different roles, interests, and influences regarding TFA elimination in Thailand. Both formal and informal communication among policy actors, and the lack thereof, aided in the policymaking process and the actions that followed. The government’s decision to impose the PHO ban was shaped by the historical, sociocultural, political, and economic contexts, and the ban was selected over other policy options to avoid the technical components of the content of a TFA elimination policy. The complexities in the policy content, context, process, and actors were attributed to the policy success of the legislated PHO ban by the FDA involving actors through Notification No. 388. In order to achieve comprehensive TFA elimination in Thailand, we recommend strengthening government actions on enforcement and raising consumer awareness. Further research on the Thai government’s progress on TFA elimination and policy analysis in different contexts is needed.

## Figures and Tables

**Table 1:** Concepts explored in interviews.

Policy Triangle Framework	Concepts	Example Questions
Content	Problematization, controversy	How was policy problematized? What controversies emerged from the selection of a particular policy problem?
Context	Historical, sociocultural, political, and economic context	How did historical, sociocultural, political, and economic contexts shape the government’s decision to impose a policy?
Process	Policymaking, policy implementation	What is the process leading to policymaking? How are policies carried out in practice?
Actors	Roles, interests, influence	What are the roles, interests, and influence of government organizations, academics, civil society organizations, and the food industry in the policy process?

**Table 2:** Description of documents in an analysis of the PHO ban in Thailand.

Author	Year	Document Name	Label
**Policy documents**			
Ministry of Public Health (MOPH)	2018	Notification of Ministry of Public Health No. 388 B.E.2561 (2018) Re: Prescribed Prohibited Food to be Produced, Imported, or Sold [22]	DOC1
Food and Drug Administration (FDA)	2018	Commentary on Notification of the Ministry of Public Health No. 388 B.E. 2561 (2018) Re: Prescribed Prohibited Food to be Produced, Imported, or Sold [23]	DOC2
FDA	2018	Implementation Manual of Notification of the Ministry of Public Health No. 388 B.E.2561 (2018) Re: Prescribed Prohibited Food to be Produced, Imported, or Sold [24]	DOC3
**Research**			
Thongurai	2012	Thai Laws, New York Laws, and the Control of Trans Fat [25]	RES1
Chavasit et al.	2018	Thailand’s Food Policy for a Trans Fat-Free Country [16]	RES2
Chavasit et al.	2019	Overcoming the Trans Fat Problem in Thailand [17]	RES3
Chavasit et al.	2020	Evolution of Trans-Fatty Acid Consumption in Thailand and Strategies for Its Reduction [18]	RES4

**Table 3:** Description of informants recruited for interviews, September 2021–January 2022.

Position/Sector	Invited Stakeholders	Number of Participants	Label
Government organizations	17	9	GO1-9
Academics	9	5	AC1-5
Civil society organizations	7	4	CS1-4
Food industry	8	2	FI1-2

## Data Availability

Not applicable.
